# Do universal codon-usage patterns minimize the effects of mutation and translation error?

**DOI:** 10.1186/gb-2005-6-11-r91

**Published:** 2005-10-19

**Authors:** Roberto Marquez, Sandra Smit, Rob Knight

**Affiliations:** 1 Department of Computer Science, New Mexico State University, MSC CS, Las Cruces, NM 88003, USA; 2 Department of Chemistry and Biochemistry, University of Colorado, Boulder, CO 80309, USA

## Abstract

**Background:**

Do species use codons that reduce the impact of errors in translation or replication? The genetic code is arranged in a way that minimizes errors, defined as the sum of the differences in amino-acid properties caused by single-base changes from each codon to each other codon. However, the extent to which organisms optimize the genetic messages written in this code has been far less studied. We tested whether codon and amino-acid usages from 457 bacteria, 264 eukaryotes, and 33 archaea minimize errors compared to random usages, and whether changes in genome G+C content influence these error values.

**Results:**

We tested the hypotheses that organisms choose their codon usage to minimize errors, and that the large observed variation in G+C content in coding sequences, but the low variation in G+U or G+A content, is due to differences in the effects of variation along these axes on the error value. Surprisingly, the biological distribution of error values has far lower variance than randomized error values, but error values of actual codon and amino-acid usages are actually greater than would be expected by chance.

**Conclusion:**

These unexpected findings suggest that selection against translation error has not produced codon or amino-acid usages that minimize the effects of errors, and that even messages with very different nucleotide compositions somehow maintain a relatively constant error value. They raise the question: why do all known organisms use highly error-minimizing genetic codes, but fail to minimize the errors in the mRNA messages they encode?

## Abstract 2

The analysis of codon usage in nearly 900 species of the three domains of life suggests that codon usage patterns in mRNA messages do not minimize the effects of translation error.

## Background

Genetic codes are arranged in a way that is highly resistant to errors, but whether the mRNAs that genomes encode also resist errors has been largely untested. The standard genetic code is found in most nuclear and mitochondrial genomes, although some genomes have slight variations in the genetic code (see [1] for review). The biochemical basis for many of these variations is known, but their purpose remains unclear. The extent to which a genetic code is resistant to errors (in replication, transcription, or translation) can be defined by an 'error value' [2,3], which is the sum of the differences in amino-acid properties when changing from each codon to each other codon that can be reached by a single-base substitution (see Materials and methods). The standard genetic code and all known variants resist error better (have a lower error value) than do random codes for a wide range of different amino-acid properties and models of random code generation [4-9], although the extent to which natural selection has reached the best of all codes remains somewhat controversial [10-13]. We now test the idea that organisms optimize their codon usage as well as their genetic code: codons with low error values might be used in preference to those with high error values, to reduce the overall probability of error.

Different organisms use the four bases in varying amounts at each of the three positions within the codon (that is, the average counts of each of the four bases in all the first positions of all the codons in a genome are different from the counts in all the second positions and the third positions) [1]. In particular, the first position is heavily biased towards purines, and the second position is somewhat biased towards A and C. These trends hold for all organisms in all three domains of life. In addition, organisms vary extensively in GC content (the fraction of bases that are G or C, as opposed to A or T) at each of the three codon positions, which also affects the amino-acid usage [1,14-16]. These features might be related to the code's error-minimizing properties: organisms might choose their codon and/or amino-acid usages in ways that reduce errors during translation [17-20].

Previous research has suggested that the GC content of a sequence can greatly affect its error-minimizing properties [20], and that amino-acid and/or codon usage may be optimized in *Drosophila *and mouse [19] but not in *Escherichia coli *[18], but no global survey has yet been performed. If mRNA messages are arranged in ways that minimize error, as has been comprehensively established for the genetic code itself (see for example [2,3,7]), this error minimization might arise by adjusting the usage of individual codons or amino acids, or by adjusting the overall base frequencies at each of the three codon positions. In particular, the error values might be especially stable against change in GC content, since organisms have mRNAs that vary over a wide range of GC content but vary little over the other two orthogonal axes of nucleotide composition. However, it is also possible that the genetic code was shaped under different selection pressures than those acting in modern organisms, resulting in codon-usage patterns that are random with respect to error minimization.

Codon and amino-acid usage statistics are now available for thousands of species from the Codon Usage Tabulated from GenBank (CUTG) database [21]. We tested whether species preferentially use codons with low error values; that is, codons that, if misread, would tend to substitute a more similar amino acid. To do this, we compared the error value of the code weighted by the actual codon usages against the error values of codes in which the codon or amino-acid usages had been randomized. Thus, we tested three specific hypotheses: first, that organisms choose codon usages that produce fewer errors than permuted or randomly chosen codon usages; second, that organisms choose amino-acid usages that produce fewer errors than permuted or randomly chosen amino-acid usages; and third, that the discrepancy in composition in the three nucleotide positions is caused by selection of codons that minimize errors in translation.

## Results and discussion

### Messages are not optimized

We used two different methods to compare the actual codon usages to randomized codon usages. First, we used 'shuffled' codon usages. In shuffled codon usages, the codons, amino acids, or positional-base frequencies were randomly permuted. This method preserves the relative frequencies of the the different codons, amino acids, or positional-base frequencies, but changes their meanings. For example, if the original amino-acid usage was 5%A, 10%G, and 2%W, the usage after shuffling might be 5%A, 2%G, and 10%W. Second, we used random codon usages that did not preserve the relative frequencies of codons, amino acids, or positional-base frequencies, but instead assigned each codon, amino acid, or positional-base frequency a random number from a uniform distribution, followed by normalization so that the frequencies summed to one (see Materials and methods). We analyzed species in the three domains of life separately: 33 archaea, 457 bacteria, and 264 eukaryotes for which at least 50 genes were available.

**Figure 1:**
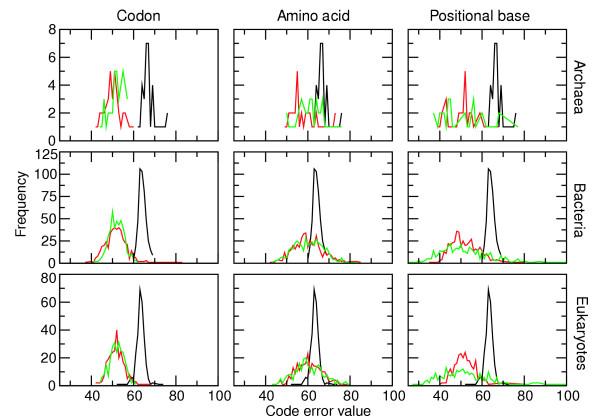
Code-error values for actual and permuted codon usages. The usages are displayed for three randomization algorithms and each domain of life. Rows: archaea, bacteria, and eukaryotes. Columns (randomization algorithms): codon, amino acid, positional base. Black, biological (unpermuted); red, permuted; green, random. Variability is always much less in the biological codon usages (black lines) than in any of the random or randomized usages, and the mean is always higher, suggesting that the biological codon usages are constrained to a narrow band but are not optimized for error minimization.

From the distributions of code-error values for real and randomized codon usages (Figure 1 first column, and Table 1), we make three observations. First, the actual distribution of error values in organisms was much tighter than in any of the randomized usages (63.8 ≤ mean ≤ 67.7 and standard deviation ≤ 3.42 for all domains). Second, both the permuted and random codon usages produced code-error values significantly lower than the corresponding values for actual codon usages (*P *≤ 0.05 by two-tailed paired *t*-test between actual and shuffled or random codon usages). Finally, the shuffled and random codon usages produced almost identical results (*P *> 0.05 in all cases by two-tailed paired *t*-test).

The variance of the actual codon usages is significantly smaller than the shuffled and random usages under each randomization model and for all domains of life. The *P*-value ranges are as follows: for archaea from 7.7 × 10^-9 ^to 0.59 (where 0.59 is the only non-significant value), for bacteria from 3.9 × 10^-257 ^to 1.1. × 10^-43^, and for eukaryotes from 8.5 × 10^-131 ^to 5.5 × 10^-10^. The significance of the difference in variance between a shuffled and random usage varies considerably (no consistent trend in *P*-values), probably depending on each specific random sample.

The pattern was similar for shuffled and random amino-acid usages, and for shuffled and random positional-base usages. In all cases, the means for the shuffled and random distributions were similar to each other and lower than the mean for the actual distribution (Figure 1, columns 2 and 3). The similarities across domains are striking: the error values for codon usages in all three domains of life fall in the same narrow region.

### Code error is not correlated with composition

To test whether the error value varied systematically with nucleotide composition, we plotted the error value as a function of position in the tetrahedron of possible base compositions (see Materials and methods for discussion). If the error value of a message depended on the composition of the codons, we would expect to see no correlation along the GC axis, because the amount of natural variation along this axis suggests that all values are selectively neutral and that therefore the code error is approximately the same. In contrast, we would expect to see increasing error values with increasing distance from the GC axis, constraining the biological variation in these other directions. However, contrary to these predictions, we find that for the real, permuted, and random positional-base usages, there are clear differences both in composition and in error at the three positions, but there is no systematic variation of error with composition.

**Figure 2:**
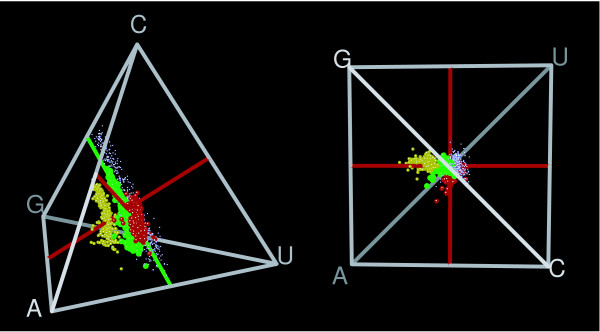
Relationship between base composition and code error. Bacterial codon usages are chosen to illustrate this relationship by plotting the base composition and code-error value for each codon position in the tetrahedral simplex (composition space). The error value for each species is plotted as a sphere with volume proportional to the error. Two perspectives are given. On the left is an oblique view to show variation along Chargaff's axis (G = C and A = T) and the relative contribution of each codon position to the error value. On the right is a view down Chargaff's axis to show the bias of each codon position. First position, yellow; second position, red; third position, blue; and total, green. As expected, the error value is always lowest at the third position (blue) as result of interconversion among synonymous codons and codons for similar amino acids.

Figure 2 shows the composition of each of the three codon positions and of the total in composition space, where the volume of a sphere is proportional to its error value. As expected, we observe clear differences in composition between the three codon positions. We can also see that the different codon positions contribute very differently to the total error value of the message. The second codon position determines about 70% of the total error value, the first codon position another 29%, and the third codon position less than 1%.

**Figure 3:**
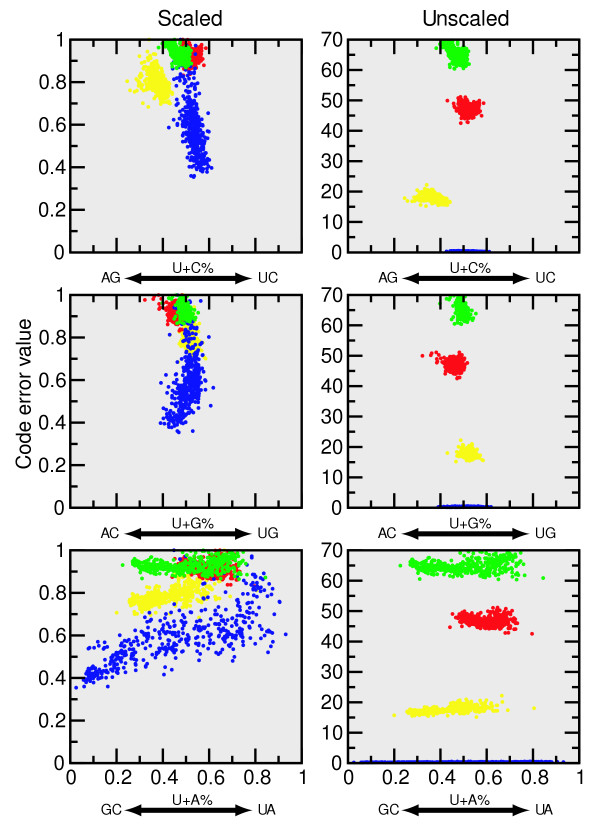
Variation in code error along the three axes in composition space: G+A, G+C, G+U. Scatterplots of variation in code-error value along each of the three axes that make up the composition space. Top row, UC content; middle row, UG content; bottom row, UA content. Left column, error value at each codon position individually scaled relative to the maximum value for that position (maximum = 1.0). Right column, absolute error values for each codon position. First position, yellow; second position, red; third position, blue; and total, green. Data shown are for bacteria, though results were similar for the other two domains (data not shown). Although substantial correlations are revealed in the scaled data, these correlations contribute little to the overall error value, which is dominated by the second codon position.

To highlight possible changes in code-error value along the three compositional axes, which are difficult to see in the simplex, we plotted code-error value versus composition along each of the three axes separately. Figure 3 shows the code-error values for the actual codon usages of bacteria along the UC, UG, and UA axes. In the left column, the error values have been scaled relative to the maximum value for each codon position independently to demonstrate relative changes, while in the right column the absolute values are displayed. Results for archaea and eukaryotes are very similar to those for bacteria (data not shown).

**Figure 4:**
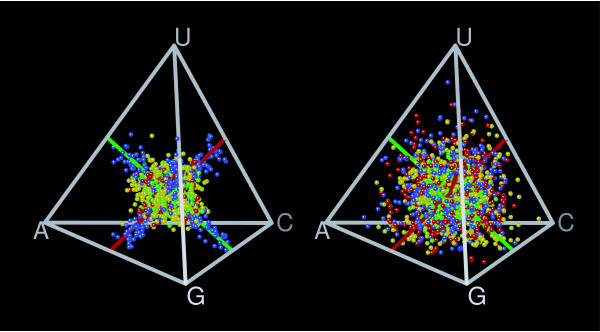
Base composition by codon position for randomized base usages. Left: permuted by positional bases, where the variability at each position is preserved, but the direction of the variability is rotated by 90 degrees around an arbitrary axis. Right: randomly chosen positional bases, where the amount of variability and the size of the correlations between axes at each position are destroyed. First position, yellow; second position, red; third position, blue; and total, green. Compare this figure with biological codon usages in Figure 2.

**Figure 5:**
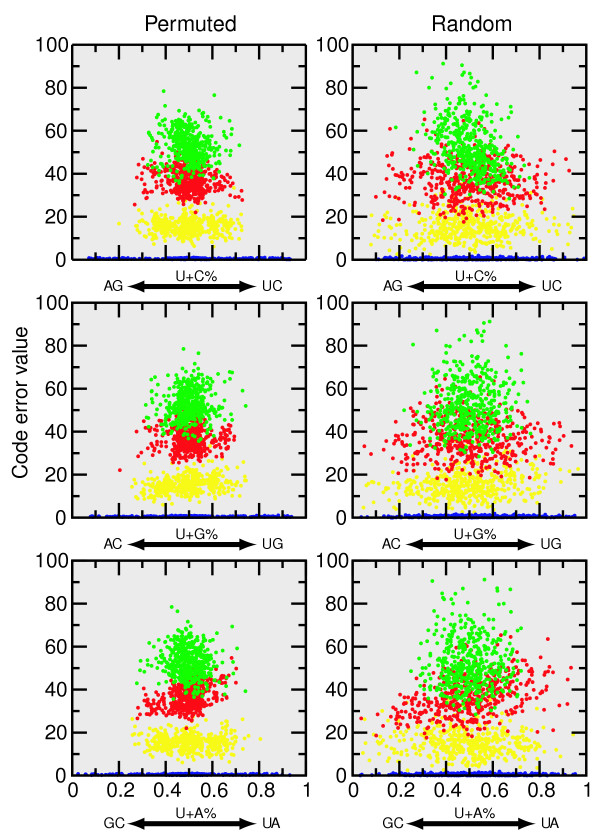
Absolute error values for permuted bacterial codon usages. The variation in code-error values is shown along the three compositional axes. Compare this figure with biological codon usages in Figure 3. Top row, UC content; middle row, UG content; bottom row, UA content. Left column, permuted positional-base usages. Right column, random positional-base usages. First position, yellow; second position, red; third position, blue; and total, green. Lack of correlation along any axis and wide range suggests that constraints on positional-base usage do not explain the pattern of codon usage error values in organisms.

We applied the same analysis to permuted and random positional-base usages, which allowed us to examine the correlations along a wider compositional range on all of the axes. These codon usages form spherical distributions around the center of the tetrahedron (Figure 4). For permuted usages, the original compositional values are redistributed over the three axes; the random usages show equal distributions for each of the three codon positions with equal variation along each axis. Figure 5 shows the corresponding scatterplots for the permuted and random usages.

We found highly significant correlations between (total) code error and position on each of the three orthogonal composition axes, except for the eukaryotes along the UG axis (Table 2). For total code error, the significant *P*-values averaged 0.0042 (range 1 × 10^-6 ^to 0.03), explaining an average of 0.19 (range 0.020 to 0.37) of the variance in code error. However, the correlation along the GC axis was not, in general, less than the correlation along the other axes. In addition, we found no significant correlations along the UG and UA axes for random and permuted data sets (in a single case the correlation was significant, but only explained 0.023 of the variation). Along the UC axis, the correlations in random and shuffled bacterial and eukaryotic usages are of similar magnitude to the correlations in the natural usages. Together with the observation that actual usage errors are typically higher than random usage errors, these observations suggest that selection against errors caused by variation along the different composition axes cannot explain observed trends in codon usage.

## Conclusion

If organisms were under strong selection to minimize errors in replication and translation, we would expect them to choose codons that are less prone to error. Consequently, we would expect that the actual codon, amino-acid, and positional-base usages would have lower error values than would permuted versions. However, we found exactly the opposite: the actual codon, amino-acid, and positional-base usages produce more errors than randomly chosen compositions.

Consequently, our hypothesis that genetic messages (as well as genetic codes) are optimized for error minimization was not supported by the data. However, the low variance in codon-usage error values in organisms suggests the intriguing alternative possibility that mRNAs are selected for a specific level of errors, rather than to minimize errors overall. Because the rate of evolution is limited by mutation, it is possible that the ability to tune the rate of protein sequence evolution by using error-prone codons has provided a selective advantage to modern organisms. Intriguingly, recent research suggests that the canonical genetic code allows target protein sequences to evolve far more rapidly than do the alternative genetic codes [22]. Codon usage may also be tuned for evolvability rather than for error minimization.

Another possible explanation for the limited variability in error-minimization properties is that the genetic code was shaped under very different selection pressures than those acting in modern organisms. Today, other factors, including directional mutation or selection for translation speed, may greatly outweigh the benefits that could be obtained by using error-minimizing codons or amino acids. However, such an explanation would predict that modern usages would be random with respect to code error, and would not predict the near constancy of error values in actual organisms. This work is consistent with the previous observations that messages within *E. coli *are not optimized for error minimization at the codon level [18] and that codon usage can greatly influence error minimization [20], and extends the analysis to a sample of over 700 bacterial, archaeal, and eukaryotic species. However, it does not confirm the observation that the amino-acid usage in some species is chosen in a way that minimizes errors [17,19]. This latter discrepancy could be due to the different sampling of genes or the different methods used to calculate the error value (single-step versus multi-step mutations).

As previously observed, we confirm that the three nucleotide positions differ greatly in nucleotide composition [1] and in error minimization [3]. However, we find no evidence for a relationship between these two properties. The universal maintenance of these patterns across species suggests that some kind of selection is involved, but the factors influencing this selection remain undefined. In particular, positional base-composition patterns orthogonal to the actual base-composition patterns, and occupying regions of composition space in which no organism has ever been observed, have errors no worse than do the actual usage patterns. This similarity strongly suggests that selection for error minimization does not play a role in keeping genomes within a narrow region of composition space. The nucleotide composition of a message has relatively little effect on its error value, suggesting that other factors maintain the systematic biases in composition at the three codon positions that are observed in all species and domains of life.

Thus, organisms do not choose their codon, amino-acid, or nucleotide composition in a way that minimizes the effects of errors. This observation is highly unexpected in light of the great extent to which the genetic code itself is arranged in an error-minimizing fashion, and suggests that some factor underlying the near-constant error values of codon usage across genomes in all three domains of life remains to be discovered.

## Materials and methods

We addressed our first and second hypotheses, that genetic messages are optimized for error minimization either at the codon or amino-acid level, by comparing the actual codon usages from organisms to first, permuted codon usages, in which the codon counts were preserved but the codons to which those counts applied were randomized, and second, to completely random codon usages. We addressed our third hypothesis, that the code error is robust to variation in GC content but not robust to other compositional variation, by examining the correlation between composition along each of the three compositional axes (GC, GU, and GA) and the code-error values for real, permuted, and random codon usages.

### Data source

We used the CUTG database as source for codon usages found in organisms [21]. We repeated the analysis separately for the three domains of life (archaea, bacteria, and eukaryotes). The species were classified according to the NCBI Taxonomy. We analyzed the 754 species for which at least 50 genes were available: 33 archaea, 457 bacteria, and 264 eukaryotes. Mitochondrial sequences were excluded.

### Calculating the error value of a message

The process of calculating an error value for a message (or codon usage) uses the basic method for calculating an error value for a genetic code [2,3], with the addition that the error value of a change from one codon to another is weighted by the frequency of the starting codon [18]. To maintain consistency with previous work [2,3], we measured the distance between amino acids using polar requirement, a measure of hydrophobicity [23].

The error value of a code is given by:



For all possible mutations *b *at each of the three codon positions *p *in all 64 codons *c*, we sum the weighted size of the change in amino-acid property, for example, hydrophobicity. The change is given by the difference in the amino-acid property of the amino acids encoded by the old and new codons, ν_*old *_- ν_*new*_, weighted by the abundance of the codon *w*_*c*_, the effect of the base position *w*_*p*_, and the probability of mutation to the new base given the codon and position *w*_*b*_|(*c*,*p*). A 'mutation' from a codon to itself does not add to the error value, because the same amino acid is present before and after the 'mutation'. Stop codons are excluded from the calculation. Codon frequencies were taken from the codon usage database or assigned at random. We used a range of transition/transversion biases from 1:1 to 10:1, although there was no qualitative effect on the results. Results shown are for a transition/transversion bias of 4:3, and equal weighting for the three base positions.

### Creating permuted and random codon usages

We can calculate the amino-acid usage and positional-base usage from a given codon usage. The frequency of an amino acid is the sum of the frequencies of each of its codons. A positional-base usage is the frequency of each of the four bases at each of the three codon positions. For example, the frequency of U at the first codon position is the sum of the frequencies of all codons that start with a U. Thus, each codon usage is associated with one unique amino-acid usage and one positional-base usage.

However, many different codon usages correspond to the same amino-acid usage. To predict the codon usage associated with an amino-acid usage, we used the assumption that all codons coding for the same amino acid occur with equal frequencies, so that each gets an equal share of the amino-acid frequency. Consequently, blocks of codons (coding for the same amino acid) are assigned the same frequency. The prediction of the frequency of a codon from a positional-base usage is calculated as the product of the positional-base frequencies of its bases at the three codon positions. This method reflects the idea that if a species were under selection for amino-acid usage only, there would be no *a priori *reason to assign different frequencies to the different codons for a given amino acid. Similarly, to predict the codon usage associated with a particular positional-base usage, we take the product of the frequency of the appropriate base at each of the three codon positions. For example, the frequency of the codon AUG is the product of the frequency of A at the first position, U at the second position, and G at the third position.

With the above transformations in mind, we can shuffle frequencies or choose random frequencies at three levels: codons, amino acids, and positional bases. After creating a permuted or random amino-acid usage or positional-base usage, we calculate the corresponding codon usage as described above (because the error value calculations require codon usages as input).

### Statistics

We used the two-tailed paired *t*-test to compare the means of the various distributions, because we examined the same sample before and after randomization. Differences in variance between the error values of the actual usages and the permuted and random usages were calculated by a two-tailed F-test.

### Visualization

The (positional) composition of the codon usages can be conveniently visualized with the program MAGE [24], using a presentation scheme in which the volume of a sphere is proportional to the error value at a particular codon position. The base frequency of a set of bases, such as a sequence of nucleotides or all bases at a particular codon position, can be visualized as a point in composition space. The base frequency is described as a vector of the fraction of each of the four bases (U, C, A, and G) in the set. These fractions form the four coordinates to describe sequence composition. When visualizing the space of all possible compositions, we only have three dimensions to work with. Three unique ways divide the four bases into sets of two, which provide an orthogonal coordinate system. The three axes are the lines where G+C equals A+U, G+U equals A+C, and G+A equals U+C. The GC (or AU) axis is also called Chargaff's axis, because it is the line where all perfectly Watson-Crick base-paired regions would reside. Composition space can thus be visualized as a tetrahedral unit simplex [25].

## Additional data files

The Python code and the raw data to perform the described code-error analysis are available as an Additional data file with the online version of this paper. Additional data file 1 is a tar archive containing the used CUTG records, separated for archaea, bacteria, and eukaryotes, the data used to produce the histograms in Figure 1, the kinemages used to produce Figures 2 and 4, and the data used to produce the scatterplots in Figures 3 and 5.

## Supplementary Material

Additional data file 1 A tar archive containing the used CUTG records, separated for archaea, bacteria, and eukaryotes, the data used to produce the histograms in Figure 1, the kinemages used to produce Figures 2 and 4, and the data used to produce the scatterplots in Figures 3 and 5. Click here for file

## Figures and Tables

**Table 1:** Error values for biological and random codon usages

	Archaea	Bacteria	Eukaryotes
Natural codon usages	67.7 ± 3.42	64.7 ± 1.77	63.8 ± 2.14
Codon permuted	52.4 ± 4.92	52.2 ± 5.15	52.7 ± 3.61
Codon random	52.6 ± 3.76	52.6 ± 3.47	52.4 ± 3.16
Amino acid permuted	61.6 ± 8.74	61.0 ± 6.95	61.1 ± 6.35
Amino acid random	61.0 ± 7.37	61.8 ± 6.96	61.7 ± 6.72
Positional base permuted	51.7 ± 6.49	52.3 ± 6.91	52.2 ± 5.44
Positional base random	52.1 ± 10.5	53.4 ± 12.6	52.1 ± 12.9

Mean ± standard deviation for each set of codon usages. The natural codon usages invariably have higher error values and lower standard deviations than any of the random or randomized codon usages: this pattern is consistent for all three domains of life.

**Table 2:** Correlations between composition and code-error value

		UC (or AG)	UG (or AC)	UA (or GC)
Bacteria	Natural	0.23 (1 × 10^-6^)	0.14 (1 × 10^-6^)	0.023 (0.0012)
	Permuted	0.017 (0.0055)	0.0020 (0.35)	0.023 (0.0011)
	Random	0.23 (1 × 10^-6^)	0.0026 (0.28)	0.00064 (0.59)
				
Eukaryotes	Natural	0.21 (1 × 10^-6^)	0.0021 (0.46)	0.12 (1 × 10^-6^)
	Permuted	0.14 (1 × 10^-6^)	0.00012 (0.86)	0.0033 (0.35)
	Random	0.20 (1 × 10^-6^)	0.0014 (0.55)	0.0069 (0.18)
				
Archaea	Natural	0.14 (0.029)	0.28 (0.0016)	0.37 (0.00017)
	Permuted	0.073 (0.13)	0.016 (0.49)	0.029 (0.34)
	Random	0.10 (0.071)	0.00056 (0.90)	0.025 (0.38)

Coefficient of determination (*r*^2^) and *P*-value for natural and representative randomized usages. Because of the much smaller sample size in archaea, the significance of the correlations is generally much lower than in the other two domains (*n *= 33 for archaea, 264 for eukaryotes, and 457 for bacteria).
